# Human papillomavirus infections in women seeking cervical Papanicolaou cytology of Durango, Mexico: prevalence and genotypes

**DOI:** 10.1186/1471-2334-6-27

**Published:** 2006-02-20

**Authors:** Luis Francisco Sánchez-Anguiano, Cosme Alvarado-Esquivel, Miguel Arturo Reyes-Romero, Margarita Carrera-Rodríguez

**Affiliations:** 1 Facultad de Medicina, Universidad Juárez del Estado de Durango (UJED). Durango, México; 2 Instituto de Investigación Científica, UJED. Durango, México

## Abstract

**Background:**

HPV infection in women from developing countries is an important public health problem. Therefore, we sought to determine the prevalences of HPV infection and HPV genotypes in a female population of Durango City, Mexico. Also to determine whether any socio-demographic characteristic from the women associated with HPV infection exists.

**Methods:**

Four hundred and ninety eight women seeking cervical Papanicolaou examination in three public Health Centers were examined for HPV infection. All women were tested for HPV DNA PCR by using HPV universal primers. In addition, all positive HPV DNA PCR samples were further analyzed for genotyping of HPV genotype 16, 18 and 33. Socio-demographic characteristics from each participant were also obtained.

**Results:**

Twenty-four out of four hundred and ninety-eight (4.8%) women were found infected by HPV. HPV genotype 16 was found in 18 out of the 24 (75%) infected women. Two of them were also coinfected by HPV genotype 18 (8.3%). In the rest 6 PCR positive women, genotyping for HPV genotypes 16, 18 and 33 were negative.

**Conclusion:**

The prevalence of HPV in women of Durango City is low; however, most infected women have high risk HPV genotype. The women who were studied showed low frequency of risk factors for HPV infection and this may explain the low prevalence of HPV infection. The high frequency of high risk HPV genotypes observed might explain the high rate of mortality for cervical cancer in our region.

## Background

Human papillomavirus virus (HPV) is the main causal factor of cervical cancer [1]. This virus is sexually transmitted and the male is the carrier. More than 100 HPV genotypes have been described and 20 of them have been associated with cervical cancer. In an international study performed in 1995 and using molecular biology techniques, HPV was found in more than 93% of cervical cancer specimens [2]. In addition, the researchers found that HPV genotypes 16, 18, 31 and 45 were found in 49.2%, 11.7%, 5% and 8%, respectively. HPV genotype 16 was more frequently observed in squamous carcinoma while genotype 18 was in adenocarcinoma. Prevalence of HPV infection in women varies substantially among countries and according to age and life style. For instance, in the United States 64% of teenagers studied were found infected and 77% of them were infected by high risk HPV genotypes [3]. Similarly, Korean prostitutes [4] and women with pre-neoplastic and neoplastic cervical lesions from the Federal District of Brazil [5] have shown prevalences as high as 47% and 43% of HPV infection, respectively. In contrast, prevalences as low as 3% to 14% have been found in married women from Barcelona, Spain [6], Amazonian women from Bolivia [7], and postmenopausal women of Iowa City [8]. In Mexico, cervical carcinoma is the most frequent cancer. In a study performed in the year 2000, as much as 20,292 new cases were reported [9]. HPV was identified as the most important agent associated with cervical intraepithelial neoplasia [10]. In addition, HPV types 16, 18 and 45 have been found in cervical tumors from the Mexican population [11,12]. Little is known about the molecular epidemiology of HPV in Durango State. Therefore, we performed a descriptive and cross sectional study in order to determine the prevalence of HPV infection, and HPV genotypes in women of Durango City, Mexico as well as to know whether any characteristic of the women is associated with HPV infection.

## Methods

### Study population

We have studied 498 women seeking for cervical Papanicolaou examination and attending three public health centers of Durango City, Mexico. One hundred and sixty six women from each health center were included in the study. Health centers were a University outpatient clinic (Institute for Scientific Research), a hospital of the Instituto Mexicano del Seguro Social (IMSS), and a hospital of the State Health Office (Secretaria de Salud). All 498 participants were enrolled consecutively from July to December 2003.

### Socio-demographic data

Socio-demographic data including age, birth place, residence place, marital status, occupation, age at start of active sexual activity, number of sexual partners, use of condom, and history of smoking from all 498 women studied were obtained.

### Cervical papanicolaou cytology

A cervical smear was obtained from each participant by using Ayre spatula and cytology brush. Papanicolaou smears were evaluated according to the Bethesda diagnosis criteria [13].

### HPV DNA PCR and HPV genotyping

A second cervical specimen was obtained with the aid of a cervical brush for HPV DNA PCR and HPV genotyping. DNA extraction was performed by using DNAzol (Invitrogen Inc. Carlsbad. CA, USA). With this method, good quality DNA was obtained inferred by the MW>20 kb for the genomic human DNA analyzed by means of 1% agarose electrophoresis, stained with ethidium bromide. In addition, the latter was confirmed by obtaining positive results for beta globin in all samples. HPV DNA PCR was carried out by using MY09/11 primers as described elsewhere [14]. The MY-PCR system has shown a sensitivity of 90% in samples containing multiple HPV types [15]. Concentrations of Mg++, primers, Taq polimerase, dNTP, tetramethylene sulfoxide, and DNA in each 50 μl reaction were 2 mM, 0.2 μM, 2 units, 0.2 mM, 2%, and 2 μg. DNA concentration used was determined based on preliminary analysis of DNA concentrations. Positive results were obtained with as low DNA concentration as 5 ngr. Thirty nine cycles of 94°C for one minute (denaturation), 55°C for two minutes (annealing), and 72°C for two and a half minutes (extension) were performed. PCR products were run in 2% agarose electrophoresis, stained with ethidium bromide, and visualized with the aid of UV light. Positive HPV DNA PCR samples were further analyzed for HPV genotyping. The presence of 3 genotypes were explored, namely HPV genotypes 16, 18 and 33. HPV genotyping PCR was carried out by using specific primers (Takara Mirus Bio Corp. Madison WI, USA) for amplification of the sequence containing E6 region of HPV 16, 18 and 33. The sequences were: forward common, 5'AAGGGCGTAACCGAAATCGGT3'; reverse 16, 5'GTTTGCAGCTCTGTGCATA3'; 18, 5'GTGTTCAGTTCCGTGCACA3', 33, 5'GTCTCCAATGCTTGGCACA3'. The amplified products correspond to 140 bp for HPV 16 and 18, and 141 bp for HPV 33. Cycling temperatures for HPV genotyping were as follows: forty five cycles of 95°C for one minute (denaturation), 57°C for one minute (annealing), and 72°C for one minute (extension).

### Ethical aspects

This study was approved by the institutional ethical committee. The purpose and procedures of the study were explained to all participants, and a written informed consent was obtained from all of them.

### Statistical analysis

Results were analyzed with the aid of the software Epi Info 6. To assess the association between the characteristics of the subjects and the infection, the crude odds ratio with a 95% exact confidence interval was used. In addition, comparison of the frequencies between groups was performed by the χ^2 ^test. A level of *P *< 0.05 was considered significant.

## Results

### Socio-demographic data

Most (87%) women were born in Durango State, and 37% were born in Durango City. All 498 women had a residence in Durango City. The mean age was 39 years (range: 20 to 72 years). Their occupations were: 60% housewives, 16.8% professionals, 16.4% employees, 4.2% factory workers, 1% students, and 0.7% other activities. Their marital status included 70% married, 3.8% widowed, 12% never married, 8% living together, and 6% divorced. History of smoking was present in 18% of the women. The mean age at start of active sexual activity was 20 years (range: 13 to 37). The mean number of sexual partners was 1.8 (range: 1 to 20), and 18% used condom during intercourse.

### Cervical papanicolaou cytology

Eleven (2.2%) out of the 498 samples showed abnormalities in the Papanicolaou examination. Table 1 shows the results of the cytological examination in women of the three public health centers studied.

**Table 1:** Overall prevalence of cervical lesions in women of three public health centers.

Cytological diagnosis	SS^a^		IMSS^b^		IIC^c^		Total		p value
	No.	%	No.	%	No.	%	No.	%	
Low-grade intraepithelial lesion	6	3.6	0	0	1	0.6	7	1.4	NS^d^
High-grade intraepithelial lesion	1	0.6	2	1.2	0	0	3	0.6	NS
Invasive carcinoma	0	0	1	0.6	0	0	1	0.2	NS
									
Total	7	4.2	3	1.8	1	0.6	11	2.2	0.021

^a^SS: Secretaria de Salud.

^b^IMSS: Instituto Mexicano del Seguro Social.

^c^IIC: Instituto de Investigación Científica.

^d^NS: not statistically significant.

### HPV DNA PCR and HPV genotyping

**Figure 1:**
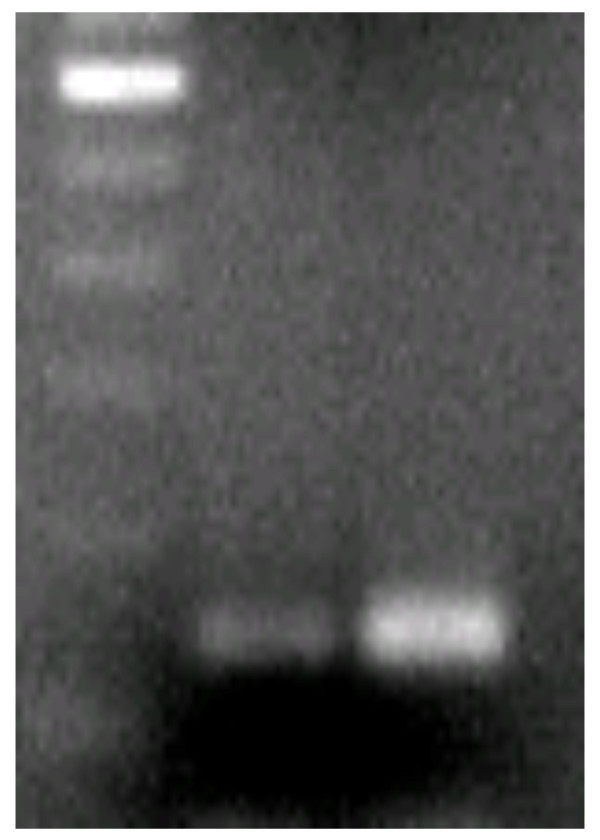
Representative gel of HPV genotyping. Left column shows a 100 bp ladder. Central and right columns show two HPV type 16 positive samples.

Women found infected by HPV were 24 out of 498 (4.8%). HPV genotype 16 was found in 18 out of the 24 (75%) infected women. Two of them (8.3%) were also infected by HPV genotype 18. While six out of the 24 HPV DNA PCR positive women showed negative results in PCR for genotyping of HPV genotypes 16, 18 and 33. A representative gel obtained in HPV genotyping is shown in Figure 1. Tables 2 and 3 show the results of the HPV DNA PCR and the distribution of HPV genotypes in the three public health centers studied, respectively. Table 4 shows a correlation of HPV genotypes and cervical lesions observed by Papanicolaou cytology.

**Table 2:** Overall prevalence of HPV infection in women of three public health centers.

HPV PCR	SS^a^		IMSS^b^		IIC^c^		Total		p value
	No.	%	No.	%	No.	%	No.	%	
Positive	18	10.8	2	1.2	4	2.4	24	4.8	0.001
Negative	148	89.2	164	98.8	162	97.6	474	95.2	NS^d^
									
Total	166	100	166	100	166	100	498	100	

^a^SS: Secretaria de Salud.

^b^IMSS: Instituto Mexicano del Seguro Social.

^c^IIC: Instituto de Investigación Científica.

^d^NS: not statistically significant.

**Table 3:** Distribution of HPV genotypes in 24 HPV PCR positive women of three public health centers.

HPV genotype	SS^a^		IMSS^b^		IIC^c^		Total		*p *value
	No.	Proportion	No.	Proportion	No.	Proportion	No.	Proportion	
16	10	0.556	2	1	4	1	16	0.667	
16 and 18	2	0.111	0	0	0	0	2	0.83	
Negative for HPV 16, 18 and 33	6	0.333	0	0	0	0	6	0.25	NS^d^
									
Total	18	0.75	2	0.83	4	0.167	24	1	

^a^SS: Secretaria de Salud.

^b^IMSS: Instituto Mexicano del Seguro Social.

^c^IIC: Instituto de Investigación Científica.

^d^NS: not statistically significant.

**Table 4:** Correlation of HPV genotypes and cytology results in 11 women with cervical lesions.

HPV genotype	LSIL^a^		HSIL^b^		IC^c^		Total		*p *value
	No.	Proportion	No.	Proportion	No.	Proportion	No.	Proportion	
16	2	0.286	2	0.667	1	1	5	0.454	NS^d^
16 and 18	1	142	1	0.333	0	0	2	0.182	NS^d^
33	0	0	0	0	0	0	0	0	NS^d^
PCR positive but negative for genotyping	2	0.286	0	0	0	0	2	0.182	NS^d^
PCR negative	2	0.286	0	0	0	0	2	0.182	NS^d^
									
Total	7	0.636	3	0.273	1	0.91	11	1	0.021

^a^Low-grade intraepithelial lesion.

^b^High-grade intraepithelial lesion.

^c^Invasive carcinoma.

^d^NS: not statistically significant.

## Discussion

In this study, we found a 4.8% prevalence of HPV infection in women seeking cervical Papanicolaou examination of Durango City, Mexico. This prevalence is lower than those reported in other regions of Mexico and the majority of those reported abroad. In two previous Mexican studies, prevalences of 14% and 15% were found [16,17]. While in a Nigerian study [18] a prevalence of 26.3% was found. The prevalence found in the present study is similar to those found in a Spanish study [6] and a Bolivian study [7] where 3% and 5.2% of women were infected, respectively. The lower prevalence found in our study than those reported elsewhere might be explained by differences in the characteristics of the study population. Nevertheless, when the prevalence of HPV infection in each health center is evaluated independently, the 10% prevalence of HPV infection in women attending the hospital of the State Health Office is closer to that reported in other Mexican States [16,17,19]. Women attending this hospital have a lower socioeconomic status than those attending the other health centers explored. In addition, those women attending the hospital with the lower prevalence (IMSS) were older than those of the other two health centers. This finding agrees with the observation that prevalence of HPV infection decreases with age. In women from Flanders with abnormal cytology, prevalences of 82% at age 22, 60% at age 47, and 52% at age 65 were found [20]. Also in our study, we found a predominant HPV genotype 16 infection. This is remarkable, since HPV genotype 16 infection is considered of high risk for cervical carcinoma [1,21]. This finding might explain why morbidity and mortality by HPV have shown irregular distribution in Mexican States. In the northern Durango State, median morbidity rates but high mortality rates have been reported [9], suggesting that our women population has higher mortality rate than that from other regions because of the high prevalence of HPV genotype 16 infection. Since 75% of HPV infected women in Durango city were infected by genotype 16, we remark the need of genotyping testing in a regular basis in order to reduce the risk for cervical carcinoma. We were unable to determine the HPV genotype in 6 (25%) out of the 24 HPV DNA PCR positive samples. We have explored only the presence of 3 HPV genotypes (16, 18 and 33) because these genotypes are considered as high risk for cervical carcinoma and more likely to be found. The high number of HPV genotypes makes our PCR approach unpractical for detection of all genotypes. However, further studies may clarify what other HPV genotypes are circulating in our region. On the other hand, PCR was a better methodology for detecting HPV infection than Papanicolaou examination. In this study, Papanicolaou cytology detected only 2 out of the 24 HPV infections. This finding stresses the need of using molecular methods to improve the detection rate. High risk HPV genotypes 16 and 18 were more frequently observed in high-grade intraepithelial lesions and invasive carcinoma. These results agree with the reported association of these genotypes with malignancy [1,18,22]. Results indicate that PCR of cervix specimens for detecting HPV infection and HPV genotyping of high risk HPV genotypes is highly recommended.

## Conclusion

We concluded that prevalence of HPV infection in women seeking cervical Papanicolaou examination in Durango City, Mexico was low. However, the high risk HPV genotype 16 is responsible for the majority of infections. Women studied showed low frequency of risk factors for HPV infection and this may explain the low prevalence of HPV infection. The high frequency of high risk HPV genotypes observed might explain the high rate of mortality for cervical cancer in our region.

## Competing interests

The author(s) declare that they have no competing interests.

## Authors' contributions

LFSA conceived and designed the study protocol, applied the questionnaires, performed the Papanicolaou examination of the women, wrote the manuscript and performed the data analysis. CAE conceived and designed the study protocol, participated in the coordination and management of the study, performed the data analysis and wrote the manuscript. MRR standardized the PCR methodology and analyzed the samples for HPV DNA detection and genotyping. MCR performed the protocol and monitored the study.

## Pre-publication history

The pre-publication history for this paper can be accessed here:



## References

[B1] Bosch FX, Lorinecz A, Muñoz N, Meijer CJ, Shah KV (2002). The causal relation between human papillomavirus and cervical cancer. J Clin Pathol.

[B2] Bosch FX, Manos MM, Muñoz N, Sherman M, Jansen AM, Peto J, Schiffman MH, Moreno V, Kurman R, Shah KV (1995). Prevalence of human papillomavirus in cervical cancer: A worldwide perspective. J Natl Cancer Inst.

[B3] Tarkowski TA, Koumans EH, Sawyer M, Pierce A, Black CM, Papp-Markowitz L, Unger ER (2004). Epidemiology of human papillomavirus infection and abnormal cytologic test results in an urban adolescent population. J Infect Disease.

[B4] Choi BS, Kim O, Park MS, Kim KS, Jeong JK, Lee JS (2003). Genital human papillomavirus genotyping by HPV oligonucleotide microarray in Korean commercial sex workers. J Med Virol.

[B5] Camara GNL, Cerqueira DN, Oliveira APG, Silva EO, Carvalho LGS, Martinsi CRF (2003). Prevalence of human papillomavirus types in woman with pre-neoplastic an neoplastic cervical lesions in the Federal District of Brazil. Mem Inst Oswaldo Cruz.

[B6] De Sanjosé S, Almirall L, Lloveras B, Font R, Díaz M, Muñoz N, Catala I, Meijer CJ, Snijders PJ, Herrero R, Bosch FX (2003). Cervical human papillomavirus infection in the female population in Barcelona, Spain. Sex Transm Dis.

[B7] Cervantes J, Lema C, Hurtado L, Andrade R, Quiroga G, Garcia G, Torricos L, Zegarra L, Vera V, Panoso W, Arteaga R, Segurondo D, Romero F, Dulon A, Asturizaga D, Hurtado-Gómez L, Sonoda S (2003). Prevalence of human papillomavirus infection in rural villages of Bolivian Amazon. Rev Inst Med trop S Paulo.

[B8] Smith EM, Ritchie JM, Levy BT, Zhang W, Wang D, Haugen TH, Tup LP (2003). Prevalence and persistence oh human papillomavirus in postmenopausal age women. Cancer Detect Prev.

[B9] Tapia-Conyer R, Kuri-Morales P, Macías-Martínez CG (2000). Registro Histopatológico de Neoplasias en México.

[B10] Gonzalez-Sánchez JL, Chavez-Brambila J, Hernández-Hernández DM, Martinez-Sánchez S, Garcia-Carranca A (2002). High and low risk human papilloma virus infection in women with CIN. Diferential characteristics. Ginecol Obstet Mex.

[B11] Lizano M, Garcia-Carranca A (1997). Molecular variants of human papillomavirus types 16, 18, and 45 in tumors of the uterine cervix in Mexico. Gac Med Mex.

[B12] Berumen J, Unger ER, Casas L, Figueroa P (1995). Amplification of human papillomavirus types 16 and 18 in invasive cervical cancer. Human Pathol.

[B13] Solomon D, Davey D, Kurman R, Moriarty A, O'Connor D, Prey M, Raab S, Sherman M, Wilbur D, Wright T, Young N, Forum Group Members; Bethesda 2001 Workshop (2002). The 2001 Bethesda System: terminology for reporting results of cervical cytology. JAMA.

[B14] Manos MM, Waldman J, Zhang TY, Greer CE, Eichinger G, Schiffman MH, Wheeler CM (1994). Epidemiology and partial nucleotide sequence of four novel genital human papillomaviruses. J Infect Dis.

[B15] Qu W, Jiang G, Cruz Y, Chang CJ, Ho GYF, Klein R, Burk R (1997). PCR detection of human papillomavirus: comparison between MY09/MY11 and GP5+/GP6+ primer systems. J Clin Microbiol.

[B16] González-Loza M del R, Laviada-Mier y Terán MA, Puerto-Solis M, García-Carrancá A (2004). Molecular variants of HPV type 16 E6 among Mexican womens with LSIL ans invasive cancer. J Clin Virol.

[B17] Calleja-Macías IE, Kalantari M, Huh J, Ortíz-López R, Rojas-Martínez A, Gonzalez-Guerrero JF, Williamson Al, Hagmar B, Wiley DJ, Villarreal L, Bernard HU, Barrera-Saldaña HA (2004). Genomic diversity of human papillomavirus 16, 18, 31 and 35 isolates in Mexican population and relationship to European, African, and Native American variants. Virology.

[B18] Thomas JO, Herrero R, Omigbodun AA, Ojemakinde K, Ajayi IO, Fawole A, Oladepo O, Smith JS, Arslan A, Muñoz N, Snijders PJ, Meijer CJ, Franceschi S (2004). Prevalence of papillomavirus in women in Ibadan, Nigeria: a population-based study. Br J Cancer.

[B19] Sánchez-Alemán MA, Uribe-Salas F, Conde-González CJ (2002). La infección por el virus del papiloma humano, un posible marcador biológico de comportamiento sexual en estudiantes universitarios. Salud Pública Mex.

[B20] Sahebali S, Depuydt CE, Segers K, Vereecken AJ, Begers JJ (2003). Cervical cytological screening an human papillomavirus DNA testing in Flanderes. Acta Clin Belg.

[B21] Berumen J, Ordoñez RM, Lazcano E, Salmerón J, Galván SC, Estrada RA, Yunes E, Garcia-Carranca A, Gonzalez-Lira G, Madrigal de la Campa A (2001). Asian-American variants of human papillomavirus 16 and risk for cervical cancer: a case-control study. J Natl Cancer Inst.

[B22] Clifford GM, Smith JS, Plummer M, Muñóz (2003). Human papillomavirus types in invasive cervical cancer worldwide: a meta-analysis. Br J Cancer.

